# *In vitro* erosive dental wear measured with a 3D intraoral scanner – a pilot study

**DOI:** 10.1080/07853890.2021.1897348

**Published:** 2021-09-28

**Authors:** Rita Gouveia, Ana Maria Vieira, José João Mendes, João Rua

**Affiliations:** a Instituto Universitário Egas Moniz (IUEM), Egas Moniz Cooperativa de Ensino Superior, Caparica, Portugal; b Centro de Investigação Interdisciplinar Egas Moniz (CiiEM), Egas Moniz Cooperativa de Ensino Superior, Caparica, Portugal

## Abstract

**Introduction:**

Erosive tooth wear is a common condition in modern societies. Without preventive measures, it can lead to dentine hypersensitivity, loss of vertical dimension of occlusion and aesthetic problems [1]. Suitable diagnostic tools need to be developed for use in prevalence studies in order to compare the results of different investigations as well as for monitoring the progression of tooth wear, as highlighted in the European Consensus statement on management guidelines for severe tooth wear [2]. The use of 3 D intraoral scans has increased, however surface alignment for comparison of sequential scans is a complex process. The goal of this *in vitro* pilot study was to develop a protocol for a future study on the accuracy of an intraoral scanner and software for the measurement of erosive wear. Focus was made on the development of a reliable method for superimposition and subtraction of intraoral scans, facilitating the measurement of wear progression using volume change.

**Materials and methods:**

Ethical approval was obtained. An extracted sound third molar was cut in the mesiodistal direction, approximately 4 mm above the cementoenamel junction. The apical part was discarded. The coronal part was bonded to a ceramic block with pre-heated composite resin and scanned at baseline with an intraoral scanner (IOS) (3 M True Definition Scanner, 3 M, USA). Erosion was simulated by immersion of the sample in the soft drink Sprite® (not degassed, pH of 2,81), under controlled agitation, 7 times 1 h. A baseline scan was made before erosion and the scans were repeated after each erosive challenge of 1 h. All measurements were made with Geomagic Control (3 D Systems, Darmstadt, Germany). The scans were imported one by one and aligned with a baseline scan. Then with all the scans aligned with the baseline, they were altogether cut at the same plane resulting in 3 D images for which was possible to determine the volume (Figure 1). Wear was calculated using volume change.

**Results:**

Calculated volume change after 1, 2, 3, 4, 5, 6, and 7 h erosion was −5.63, 3.48, 0.02, −2.48, −3.32, −1.62 and −3.12 mm^3^, respectively. Macroscopic enamel demineralisation was observed with the naked eye after 2 h erosion. Areas of enamel loss were clearly observed in the scans and seemed to increase in area and depth with increasing erosion time (Figure 2).

**Discussion and conclusions:**

Under the limitations of this pilot study, it can be concluded that the present protocol allows enamel loss detection with the IOS, and that the customised alignment of the scans is necessary for reliable volume measurements.

**Figure 1.**
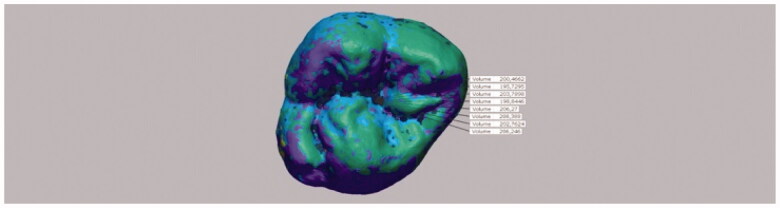
Volume determination.

**Figure 2.**
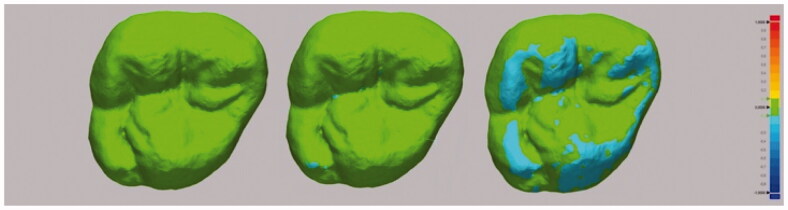
3D comparison at the baseline, 4 h and 7 h.
